# In situ imaging of microstructure formation in electronic interconnections

**DOI:** 10.1038/srep40010

**Published:** 2017-01-12

**Authors:** M. A. A. Mohd Salleh, C. M. Gourlay, J. W. Xian, S. A. Belyakov, H. Yasuda, S. D. McDonald, K. Nogita

**Affiliations:** 1 Nihon Superior Centre for the Manufacture of Electronic Materials (NS CMEM), School of Mechanical and Mining Engineering, The University of Queensland, 4072 St Lucia, Queensland, Australia; 2 Centre of Excellence Geopolymer and Green Technology, School of Materials Engineering, Universiti Malaysia Perlis (UniMAP), Taman Muhibbah 02600, Jejawi, Arau, Perlis, Malaysia; 3 Department of Materials, Imperial College, London SW7 2AZ, United Kingdom; 4 Department of Materials Science and Engineering, Kyoto University, Sakyo-ku, Kyoto 606-8501, Japan

## Abstract

The development of microstructure during melting, reactive wetting and solidification of solder pastes on Cu-plated printed circuit boards has been studied by synchrotron radiography. Using Sn-3.0Ag-0.5Cu/Cu and Sn-0.7Cu/Cu as examples, we show that the interfacial Cu_6_Sn_5_ layer is present within 0.05 s of wetting, and explore the kinetics of flux void formation at the interface between the liquid and the Cu_6_Sn_5_ layer. Quantification of the nucleation locations and anisotropic growth kinetics of primary Cu_6_Sn_5_ crystals reveals a competition between the nucleation of Cu_6_Sn_5_ in the liquid versus growth of Cu_6_Sn_5_ from the existing Cu_6_Sn_5_ layer. Direct imaging confirms that the β-Sn nucleates at/near the Cu_6_Sn_5_ layer in Sn-3.0Ag-0.5Cu/Cu joints.

Microstructures are an important link between materials processing and performance, and microstructure control is essential for any materials processing route where the microstructure plays a major role in determining the properties^1^. In the application of electrical and electronic devices, Sn alloys are used widely as solder materials, joining one electrical connection to another. The interconnection microstructure can strongly affect the performance of a joint including the fatigue life during thermal cycling^2^, the tolerance to drop impacts^3^ and the resistance to electromigration^4^. Hence, microstructure formation in solder joints is an important area of research^5^,^6^ particularly considering the need to develop more advanced electronic joining technologies while meeting the global environmental demands required of Pb-free solder joints^7^,^8^.

In the last decades, characterization techniques for understanding microstructure development in a solidification process have advanced significantly with the development of 3rd generation synchrotron X-ray imaging techniques. This has enabled studies of Sn alloy solidification related to columnar and equiaxed dendritic growth, eutectic solidification, intermetallic growth behaviour and stresses and strain induced by phase transformations^9^,^10^,^11^,^12^,^13^,^14^,^15^,^16^. Furthermore, several studies have focussed on X-ray imaging of solder solidification during a solder reaction with a substrate such as Cu. In our previous study^17^,^18^, we reported on the formation of the Cu_6_Sn_5_ interfacial layer at the liquid/Cu interface during the early stages of soldering and the effect of Ni on the growth of primary (Cu,Ni)_6_Sn_5_ in Sn-0.7Cu/Cu joints. In synchrotron studies of soldering solidification (with a substrate), Ma *et al*.^19^ and Huang *et al*.^20^ reported the observation of a morphology change of large Ag_3_Sn plates in Sn-3.5Ag/Cu joints. In addition, Qu *et al*.^21^ investigated thick interfacial Cu_6_Sn_5_ layer growth at a late stage of soldering at 350 °C. Qu *et al*.^22^ in another study investigated interfacial flux void formation in pure Sn and Sn-3.5Ag foils soldered on Cu. However, from existing synchrotron studies of solder reactions, the solder joint experiments were conducted at low frame rates (above 0.5 s per frame) and fast reactions during the soldering process, especially in the early stages of reactive wetting and subsequent solidification, were unable to be investigated. Previous synchrotron soldering experiments also used large volumes of solder and Cu foils as substrates, and small volumes of solder paste and substrates with a surface finish similar to industrial soldering have not been reported.

Although there have been many studies of microstructure formation in Pb-free solder joints by post mortem methods^23^,^24^,^25^,^26^, outstanding questions remain on how solder joint microstructures develop in the soldering process that are difficult to conclusively answer using these techniques. For example, the nucleation and growth of flux voids in the solder paste during activation and early wetting and their interaction with solidification reactions during soldering are not well understood. There is limited information on the nucleation time of the interfacial Cu_6_Sn_5_ intermetallic layer and interfacial voids, of the kinetics of primary Cu_6_Sn_5_ solidification in solder joints, and of the β-Sn dendrite nucleation location in joints.

Here we conduct a synchrotron radiography investigation on the reflow of commercial solder pastes on FR-4 type printed circuit boards (PCBs), with Cu-plating and organic soldering preservative (OSP) surface finish, that mimics the solder reaction and solidification processes that occur in electronics manufacturing. This approach enables the direct observation and quantification of flux activation, solder paste melting, reactive wetting and flux void formation during heating to the peak temperature and then the nucleation and growth of primary intermetallic compounds (IMCs) and the point of β-Sn nucleation during solidification. We use Sn-3.0Ag-0.5Cu and Sn-0.7Cu (wt.%) solder pastes as case studies with a particular focus on quantifying the nucleation and growth kinetics of flux voids and primary Cu_6_Sn_5_ crystals, understanding their formation mechanisms, and identifying the location of β-Sn nucleation.

## Results

### Reactive wetting and flux void development

**Figure 1:**
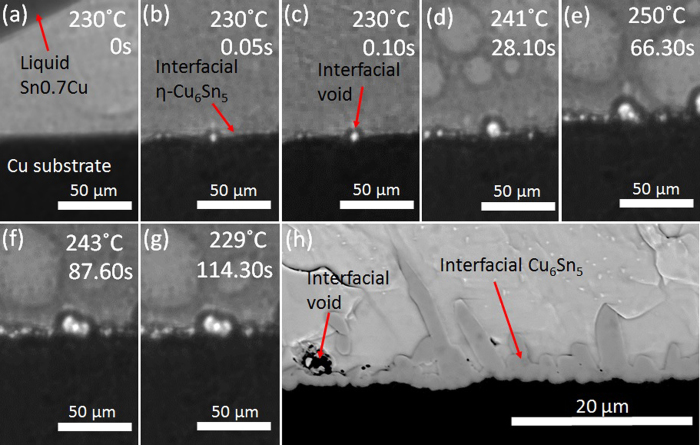
*In situ* real time imaging observations of interfacial Cu_6_Sn_5_ formation during early wetting reactions between liquid Sn-0.7Cu and the Cu substrate interface at (**a**) 0 s, (**b**) 0.05 s, (**c**) 0.10 s, (**d**) 28.10 s, (**e**) 66.30 s, (**f**) 87.60 s and (**g**) 114.30 s times after wetting: (**h**) a post mortem backscattered electron scanning electron microscopy (SEM) image of the solidified sample.

A typical example of the first moments of solder wetting and spreading is shown in Fig. 1a–g for molten Sn-0.7Cu on Cu. Initially, in Fig. 1a, the liquid is not in contact with the Cu substrate and, between the frames in Fig. 1a and b, the liquid spreads from left to right over the Cu. Within 0.05 s of solder wetting, the dissolution of Cu from the substrate caused the formation of a η-Cu_6_Sn_5_ layer. Together with the formation of interfacial layer, interfacial voids were also present (Fig. 1b). Subsequently, the layer develops a scalloped interface and the growth of interfacial solder voids is observed. Figure 1h is a post- mortem scanning electron microscopy image of the same sample which shows the interfacial Cu_6_Sn_5_ layer and interfacial voids which formed during reactive wetting.

**Figure 2:**
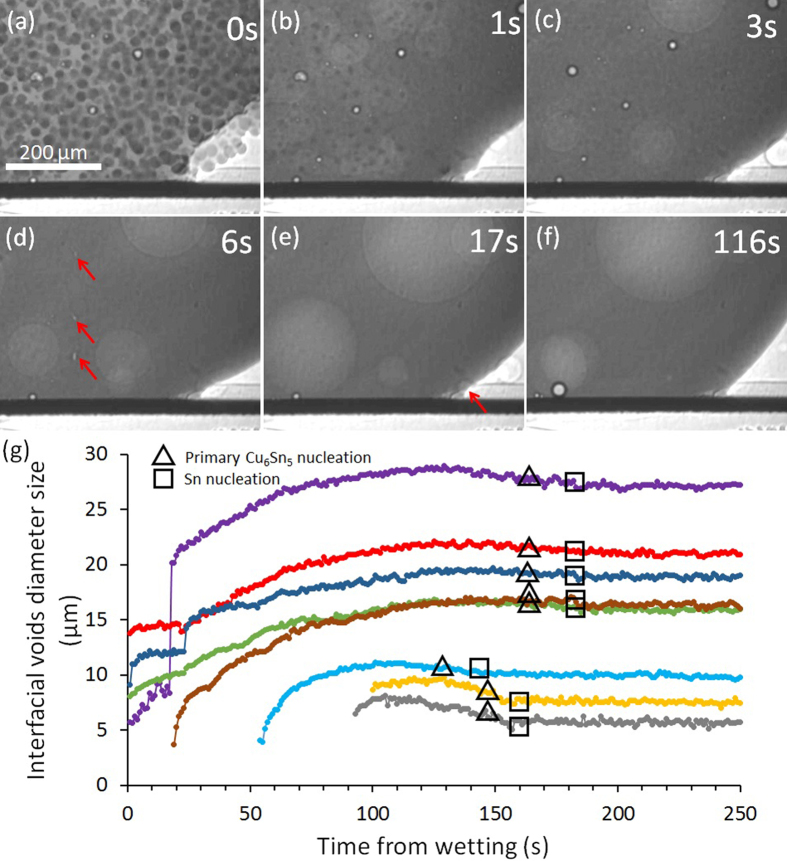
(**a–f**) Synchrotron image sequence of the flux void formation during early wetting of Sn-0.7Cu paste on a Cu substrate and (**g**) quantification of interfacial voids size (diameter) formation and growth in Sn-0.7Cu paste during soldering from synchrotron image sequences (compilation from several experiments where different colours indicates different flux voids).

Figure 2a shows the solder paste on the Cu-plated PCB at the first moment of solder melting. The individual ~35 μm grains of solder powder can be seen suspended in flux. Figure 2a shows the first moment of melting. There are a number of voids/bubbles in the paste and at the paste-Cu interface which exist from the first moments of melting. These are mostly caused by flux outgassing^17^,^24^. At the stage shown in Fig. 2a, the voids have an irregular shape and are present between the solid solder grains. As the solder paste fully melts in Fig. 2a,b and c, the voids develop into a spherical shape to minimise their interfacial area with the molten solder. Figure 2b is a snapshot during solder paste melting where both liquid solder and unmelted solder powder coexist. In the first few seconds after melting (Fig. 2b–d), many flux voids first become more spherical and then float up and out of the solder ball due to buoyancy. However, comparing Fig. 2b–f, it can be seen that the flux voids at the solder-substrate interface do not float upwards despite being significantly less dense than the liquid solder.

A bubble is expected to remain attached to the Cu_6_Sn_5_ layer if the balance of interfacial energies satisfies the following inequality (assuming for simplicity that there is only a small contact area between the bubble and interfacial intermetallic compound (IMC) so that the bubble shape is unchanged):


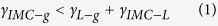


where γ is the interfacial Gibbs energy per unit area (IMC-g refers to intermetallic compound layer and flux void interface, L-g refers to liquid solder and flux void interface and IMC-L refers to intermetallic compound layer and liquid solder interface). This inequality is likely to be met given the relatively high interfacial energy between liquid Sn and Cu_6_Sn_5_. The total interfacial energy would be even lower if bubbles were located in the grooves between the Cu_6_Sn_5_ scallops. Therefore, they would be partially stabilised against a buoyancy force in these locations. This appears to be the origin of the difficulty of removing flux voids from the solder-substrate interface. It can also be seen in Fig. 2e,f, that the size of the interfacial flux voids increases with time and that new voids nucleate and grow at the Cu_6_Sn_5_-L interface between 17 and 116 s after the onset of melting. Figure 2g is a compilation plot showing the diameter of interfacial flux voids as a function of the time from wetting. Note that the measurements were gathered from several Sn-0.7Cu/Cu synchrotron imaging experiments. From this plot, it can be seen that some interfacial flux voids of 5–15 μm were observed from the moment of wetting and that further flux voids nucleate later as the joint is heated towards the peak reflow temperature. All observable interfacial voids grow rapidly to a size of at least 4 μm in diameter and then continue to expand (maximum expansion near peak temperature ~250 °C) and then contract during cooling. Towards the end of contracting, a sudden slight increase (approximately 1%) in their size was observed coinciding with the β-Sn nucleation event, which is associated with solidification shrinkage as the undercooled liquid solidifies within a few frames. Subsequently, it can be seen that the flux void sizes remain constant after β-Sn nucleation when they are surrounded by solid.

**Figure 3:**
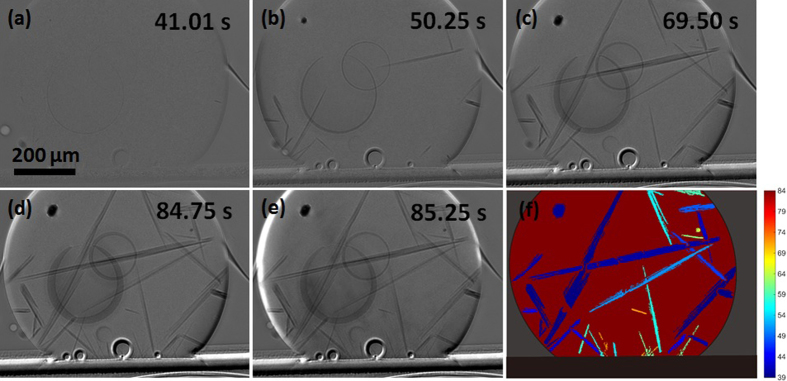
Synchrotron image sequence of the nucleation and growth of primary Cu_6_Sn_5_ in a Sn-3.0Ag-0.5Cu/Cu joint. Images have been normalised against a frame shortly before that in (**a**). Cu_6_Sn_5_ are dark. The two central round features are bubbles. The round features at the Cu_6_Sn_5_ interfacial layer are flux voids. (**f**) a processed image with each Cu_6_Sn_5_ segmented and coloured by its nucleation time in s. t=0 is the onset of cooling from the peak temperature of 250 °C.

**Figure 4:**
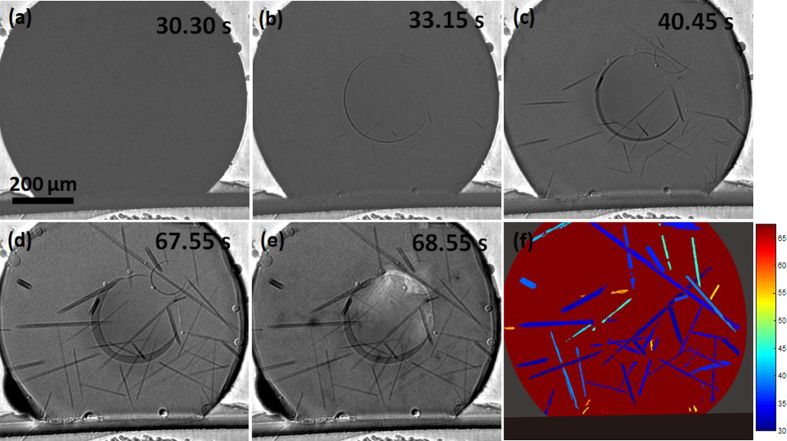
Synchrotron image sequence of the nucleation and growth of primary Cu_6_Sn_5_ in a Sn-0.7Cu/Cu joint. Images have been normalised against a frame shortly before that in (**a**). Cu_6_Sn_5_ are dark. The central round feature is a bubble. The round features at the Cu_6_Sn_5_ interfacial layer are flux voids. (**f**) is a processed image with each Cu_6_Sn_5_ segmented and coloured by its nucleation time in s. t=0 is the onset of cooling from the peak temperature of 250 °C.

Similar interfacial flux voids formed in all experiments, as can be seen in Figs 1, 3 and 4. In some experiments, bubbles of evaporated flux continuously formed near the air-solder-substrate triple points, were transported upwards along the sides of the solder ball and then floated away. Examples of this are highlighted with arrows in Fig. 2e. Note that the large round features near the centre of the solder balls in Figs 1, 2, 3, 4 are shallow bubbles between the sample and the quartz confining sheets. These are artifacts of the experiment and form even without flux and will not be discussed further.

### Nucleation and growth of primary Cu_6_Sn_5_ crystals

Figs 3a–e and 4a–e, show the development of primary Cu_6_Sn_5_ during continuous cooling from the peak temperature of 250 °C in Sn-3.0Ag-0.5Cu/Cu and Sn-0.7Cu/Cu respectively. On both figures, the central round features are shallow bubbles (artifacts) while the round features at the Sn/Cu_6_Sn_5_/Cu interface are flux voids. The dark rods in the solder ball are primary Cu_6_Sn_5_ and the tin liquid is slightly brighter. Figure 3f and 4f are processed images where each Cu_6_Sn_5_ crystal has been segmented and colored by its nucleation time to visualise the sequence of nucleation events. In Sn-3.0Ag-0.5Cu/Cu, many Cu_6_Sn_5_ grew from the edge (the side surface) of the solder ball into the liquid as observed in Fig. 3a–c and in Fig. 3f (dark blue). Other Cu_6_Sn_5_ crystals appear to nucleate in the bulk liquid but note that there are also solder surfaces perpendicular to the x-ray beam. The Cu_6_Sn_5_ nucleation location can be inferred from the observation that the crystals did not move under gravity despite being significantly denser than liquid Sn (8,082 vs 6,967 kg/m^3^ at 250 °C^27^,^28^). This suggests that the Cu_6_Sn_5_ crystals nucleated on the surface or on particles attached to the surface (possibly on the oxide where the SnO-L interfacial energy is relatively high) in both Sn-3.0Ag-0.5Cu/Cu and Sn-0.7Cu/Cu joints. Also it can be observed from the colour maps in Figs 3f and 4f that the nucleation sequence of primary Cu_6_Sn_5_ in both materials bears no detectable relationship to the small temperature gradient of ~1 K (from the top to bottom of the field of view) and nucleation events do not follow an isotherm sweeping through the sample.

**Figure 5:**
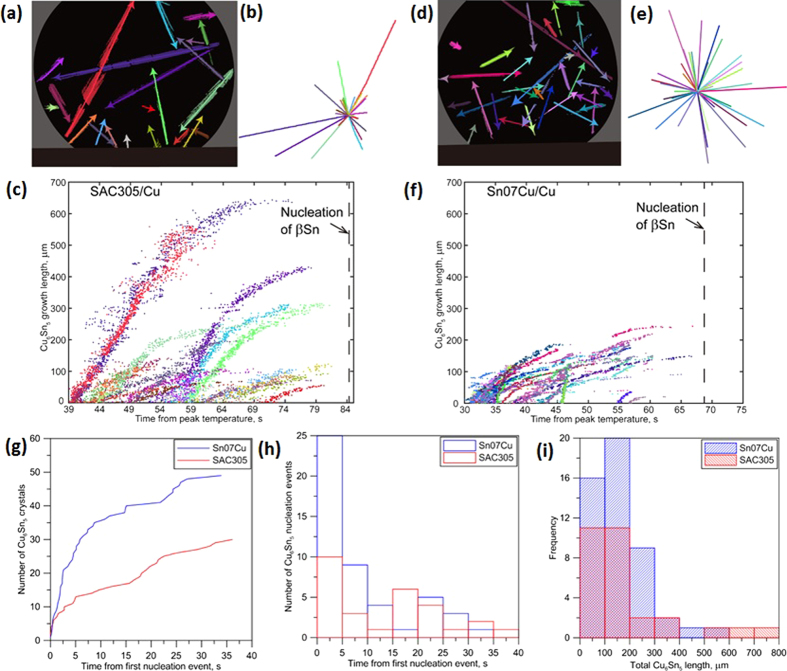
Quantification of the solidification kinetics of primary Cu_6_Sn_5_ crystals from the synchrotron image sequences in Figs 3 and 4. (**a–c**) Sn-3.0Ag-0.5Cu/Cu. (**d–f**) Sn-0.7Cu/Cu. (**a**) and (**d**): Cu_6_Sn_5_ growth vectors. (**b**) and (**e**): the same vectors with a common origin showing the growth orientation distribution. (**c**) and (**f**): the growth tip position versus time for most Cu_6_Sn_5_ crystals in each sample. (**g**) total number of Cu_6_Sn_5_ crystals versus time for each joint. (**h**) histogram of the number of Cu_6_Sn_5_ nucleation events with time. (**i**) size distribution of the primary Cu_6_Sn_5_ crystals at the end of solidification in both joints.

The solidification kinetics of all primary Cu_6_Sn_5_ crystals in a joint of Sn-3.0Ag-0.5Cu/Cu and Sn-0.7Cu/Cu are quantified in Fig. 5. Since the primary Cu_6_Sn_5_ crystals grew as faceted rods without branching, their growth could be quantified by a single vector. Fig. 5a-5d are plots of Cu_6_Sn_5_ growth vectors and Fig. 5b-5e are similar plots using a single origin. They show that there is no preferred Cu_6_Sn_5_ growth direction which is consistent with growth from randomly oriented nucleation sites.

Figure 5c-5f are plots of the growth length of primary Cu_6_Sn_5_ crystals as a function of time, where Fig. 5a,b and c have a common Cu_6_Sn_5_ colour scale as do Fig. 5d,e and f. Here, ‘growth length’ is the distance from the nucleation point to the growth tip along the main [0001] growth direction. In the Sn-3.0Ag-0.5Cu/Cu joint, the first two Cu_6_Sn_5_ crystals grew with a near-constant tip velocity of 9.8 μm/s for ~600 μm before slowing down due to solute field interaction with surrounding growing Cu_6_Sn_5_ crystals. Most other Cu_6_Sn_5_ crystals in Fig. 5c exhibited nonlinear growth from immediately after nucleation because the existing Cu_6_Sn_5_ led to overlapping solute fields reducing the tip undercooling. In the Sn-0.7Cu/Cu joint, there was little linear growth (Fig. 5f) because crystals nucleated in close proximity and solute fields overlapped early during growth.

**Figure 6:**
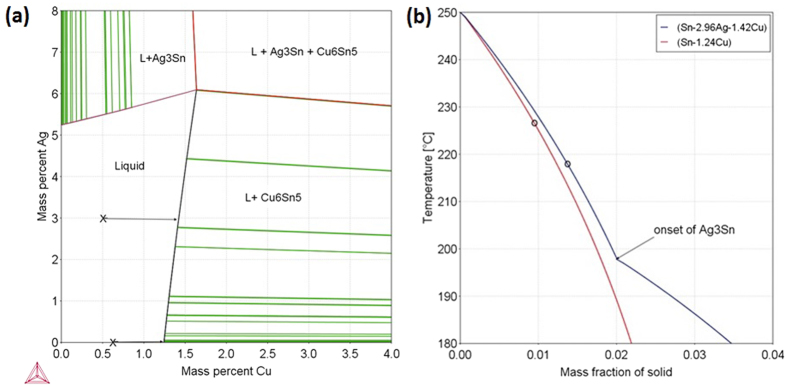
(**a**) Sn-Ag-Cu 250 °C isothermal section based on Thermocalc^29^. Crosses mark the initial solder compositions and the arrows show the change in liquid composition due to Cu substrate dissolution (up to the solubility limit). (**b**) Predicted development of primary IMC during cooling from 250 °C in Sn-3.0Ag-0.5Cu/Cu and Sn-0.7Cu/Cu joints, assuming the liquid was saturated in Cu at 250 °C. The circles show the equilibrium eutectic onset temperatures and, below these temperatures, primary Cu_6_Sn_5_ only continues to grow if β-Sn fails to nucleate. Note that, in Sn-3.0Ag-0.5Cu/Cu, primary Ag_3_Sn crystals are not predicted to form until ~198 °C (ΔT_nuc_~19 K).

Fig. 5g shows the number of Cu_6_Sn_5_ crystals versus time, Fig. 5h is a plot of the number of Cu_6_Sn_5_ nucleation events versus time, and Fig. 5i shows the distribution of final lengths of Cu_6_Sn_5_ crystals for both joints. Combining Fig. 5g–i, it is clear that Cu_6_Sn_5_ rods are more numerous and, generally, shorter in Sn-0.7Cu/Cu joints than in Sn-3.0Ag-0.5Cu/Cu joints. Some understanding of the origin of more primary Cu_6_Sn_5_ nucleation events in Sn-0.7Cu/Cu can be gained from the predicted solidification path in Fig. 6, which assumes that dissolution of the substrate occurs until the liquid solder is uniformly saturated in Cu. It can be seen that a slightly higher fraction of primary Cu_6_Sn_5_ is predicted to form in Sn-3.0Ag-0.5Cu/Cu than in Sn-0.7Cu/Cu for the same β-Sn nucleation undercooling. The growth restriction factor (GRF)^29^ can be deduced directly from the T vs f_s_ plots because it can be expressed as


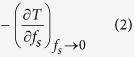


Since the T vs f_s_ slope is slightly steeper near f_s_=0 in Sn-0.7Cu/Cu, it can be seen that the GRF is higher for this joint. Thus, a given level of constitutional supercooling develops in a shorter Cu_6_Sn_5_ growth distance in Sn-0.7Cu/Cu compared with Sn-3.0Ag-0.5Cu/Cu which would enable nucleation events to occur closer together in Sn-0.7Cu/Cu.

Note that the only primary intermetallic phase observed in this work was Cu_6_Sn_5_ (i.e. Ag_3_Sn primary crystals were never observed in Sn-3.0Ag-0.5Cu/Cu joints). This can be understood from the predicted solidification path in Fig. 6: primary Ag_3_Sn are not predicted to form until below ~198 °C (ΔT_nuc_~19 K). For joints of the size studied in this work soldered to Cu, it is common for the nucleation undercooling for β-Sn to be less than 19K^30^. This interpretation was confirmed in our laboratory studies in which primary Ag_3_Sn only formed in Sn-3.0Ag-0.5Cu/Cu joints at undercoolings higher than 20 K (refer to supplementary data).

In Fig. 5h, the highest nucleation rate occurs at the beginning of primary Cu_6_Sn_5_ solidification. However, in both joints, the number of Cu_6_Sn_5_ crystals continuously increases during cooling from the peak temperature almost until the nucleation of β-Sn. That is to say, nucleation occurred continuously during cooling and did not only occur in the first stages of cooling as commonly occurs in the solidification of alloys^31^. One reason for this appears to be the highly anisotropic faceted growth mechanism of Cu_6_Sn_5_, where this crystal only grows along [0001] and does not branch (under the conditions of the paper). Thus, Cu_6_Sn_5_ cannot grow into liquid regions that are not in the [0001] growth path and constitutional supercooling builds up in these liquid regions until it exceeds the required nucleation undercooling for Cu_6_Sn_5_, when a new nucleation event is triggered. This effect is compounded by the high GRF of Cu_6_Sn_5_ in Sn-rich compositions which causes a large constitutional supercooling to develop in a relatively short growth distance. Thus, due to the combined high growth anisotropy and GRF, it is easier for new Cu_6_Sn_5_ crystals to nucleate in the liquid than it is for existing Cu_6_Sn_5_ to branch during growth, which causes continuous nucleation during cooling in a near-uniform thermal field.

**Figure 7:**
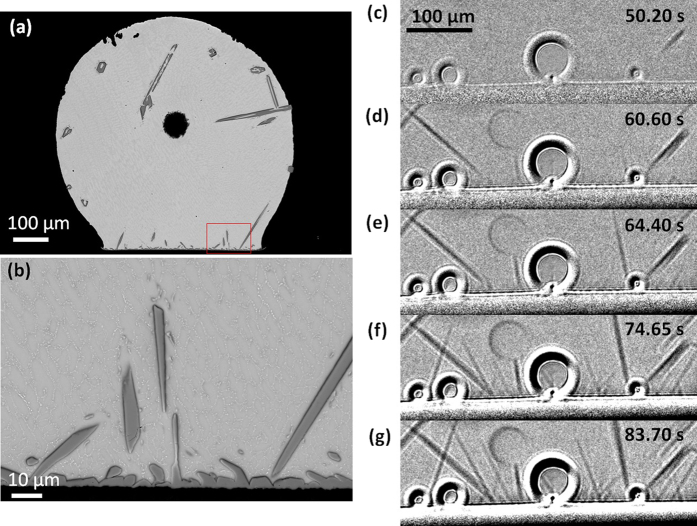
Growth of primary Cu_6_Sn_5_ on Sn-3.0Ag-0.5Cu/Cu near the interfacial intermetallic compound (IMC) layer later during solidification (**a–b**) post-mortem SEM images. (**c–g**) *in-situ* synchrotron images at five different times.

In some samples, some primary Cu_6_Sn_5_ also grew from the pre-existing Cu_6_Sn_5_ reaction layer as shown in Fig. 7c–g for Sn-3.0Ag-0.5Cu/Cu. This shows that there is competition between primary Cu_6_Sn_5_ nucleation in the liquid versus Cu_6_Sn_5_ growth from the Cu_6_Sn_5_ reaction layer. For example, as shown in Fig. 3, late during solidification of the Sn-3.0Ag-0.5Cu/Cu between 50.25 s–84.75 s of cooling time, some Cu_6_Sn_5_ crystals grew upwards from the pre-existing Cu_6_Sn_5_ layer (turquoise and orange crystals in Fig. 5(c) and (f)). Note also that other Cu_6_Sn_5_ crystals grew from elsewhere down into the layer in the same time period and, from the post mortem SEM images in Fig. 7a and b, it is often not possible to deduce whether primary Cu_6_Sn_5_ grew into the layer or grew out from the layer, which highlights the importance of *in-situ* imaging.

The growth of Cu_6_Sn_5_ out from the layer produces long IMC protrusions that are undesirable and potentially harmful to reliability. In Sn-Ag-Cu/Cu-OSP solder joints after drop impact testing, Pang^32^ reported that the crack path could occur either near the Cu-Cu_6_Sn_5_ interface, the Sn-Ag-Cu-Cu_6_Sn_5_ interface, or in the middle of the Cu_6_Sn_5._ After 500 cycles of thermal cyclic testing, drop impact tests indicated that the dominant cracking was observed in the Cu_6_Sn_5_ interfacial layer^32^. In addition, Tian *et al*.^33^ have proven that long primary Cu_6_Sn_5_ crystals in the bulk solder joint were the crack sources during *in-situ* tensile tests of Sn-3Ag-0.5Cu/Cu after multiple reflows. Thus, it is important to understand the conditions under which Cu_6_Sn_5_ grows out from the layer and methods to prevent it. It was found that samples where Cu_6_Sn_5_ grew out from the layer usually had a region of open liquid ahead of the layer relatively late during primary Cu_6_Sn_5_ solidification. Open liquid regions remain when no or few primary Cu_6_Sn_5_ rods have their [0001] growth direction oriented towards the IMC layer (e.g. Fig. 3). In this situation, new Cu_6_Sn_5_ can only nucleate in the liquid ahead of the layer if the constitutional supercooling in this region exceeds the required nucleation undercooling. This is less likely in the solute field ahead of the growing Cu_6_Sn_5_ layer than in the liquid far from the layer, and becomes even less likely late during cooling when the solute fields of surrounding primary Cu_6_Sn_5_ have impinged on the solute field of the Cu_6_Sn_5_ layer. When the nucleation of new primary Cu_6_Sn_5_ crystals are suppressed in this way, the conditions exist for Cu_6_Sn_5_ to grow out from the existing Cu_6_Sn_5_ layer. The development of open liquid regions ahead of the Cu_6_Sn_5_ layer is more likely when there are few, large Cu_6_Sn_5_ rods (such as in Fig. 3) because this increases the chance that a few Cu_6_Sn_5_ rods will have a [0001] direction towards the layer. On the other hand, Cu_6_Sn_5_ can be prevented from growing out from the layer by encouraging numerous small Cu_6_Sn_5_ to nucleate throughout the liquid (with numerous growth directions) so that no large liquid region develops ahead of the interfacial IMC layer into which layer crystals need to grow (e.g. for Sn-0.7Cu/Cu in Fig. 4a–f).

### β-Sn nucleation and growth

**Figure 8:**
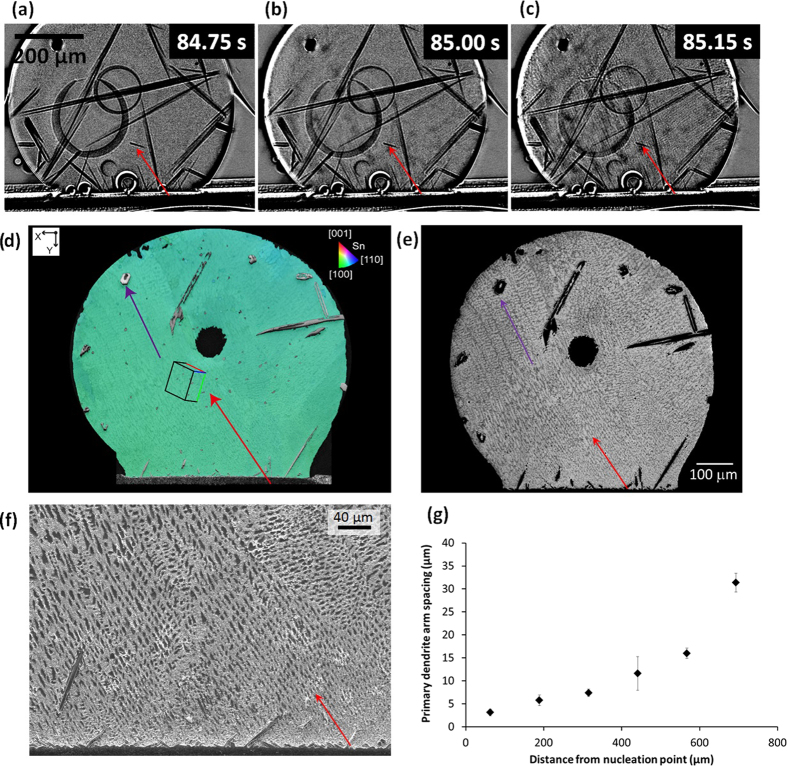
(**a–c**) Synchrotron images of the nucleation and growth of β-Sn in the Sn-3.0Ag-0.5Cu/Cu joint. (**d**) EBSD inverse pole figure (IPF)-y map of the Sn-3.0Ag-0.5Cu/Cu joint with the unit cell orientation superimposed. (**e**) BSE-SEM image in which the β-Sn dendrite growth direction can be deduced. Arrows show the growth direction in two regions projected onto the imaging plane. (**f**) BSE-SEM image of dendrites near the nucleation site. (**g**) plot of primary dendrite arm spacing versus distance from the observed nucleation point.

Although the *in-situ* imaging technique used here was not optimized for the high interface velocities after β-Sn nucleates, useful information could still be extracted. The nucleation location of β-Sn in Sn3.0Ag0.5Cu/Cu joints was observed to be at/near the interfacial Cu_6_Sn_5_ layer as indicated by the red arrow towards the top (Fig. 8a–c). This has been inferred in previous ‘post mortem’ work^30^,^34^ but here we directly prove that β-Sn nucleates on or near the Cu_6_Sn_5_ layer in Sn-3.0Ag-0.5Cu/Cu joints. The subsequent growth of the β-Sn dendrite can be seen by comparing Fig. 8a,b and c where the dendrite growth velocity was measured to be around 800 μm/s early during growth. Post-mortem EBSD mapping in Fig. 8d shows that there is a single β-Sn crystal in the joint. Comparing the EBSD inverse pole figure (IPF) map with the BSE-SEM image in Fig. 8e shows that the dendrite growth direction is close to <110>. With the knowledge of the nucleation location (the start of the red arrow in Fig. 8a–f), the β-Sn primary dendrite arm spacing was measured as a function of growth distance. The primary dendrite arm spacing increases from around 10 μm at the nucleation location to 300 μm at the maximum distance from nucleation (Fig. 8g), which is consistent with the dendrite tip velocity decreasing during growth into an undercooled melt due to the release of latent heat and consequent decrease in tip growth undercooling^24^,^35^,^36^. Previous research has shown that Sn-3.0Ag-0.5Cu/Cu joints usually solidify with one β-Sn orientation or two or three β-Sn orientations that are all related by a twin orientation relationship^30^,^34^,^37^,^38^. The presence of a single β-Sn orientation in Fig. 8 is consistent with this.

**Figure 9:**
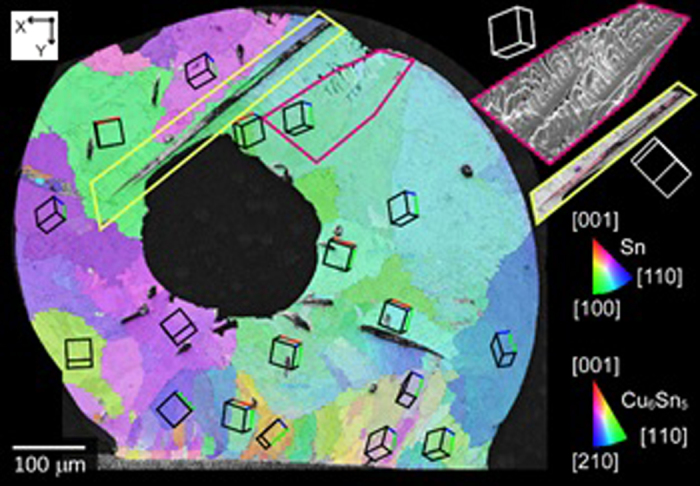
EBSD inverse pole figure (IPF)-y map of the Sn-0.7Cu/Cu joint with unit cell orientations superimposed. The inserts show the β-Sn dendrite morphology relative to its unit cell orientation, and a primary Cu_6_Sn_5_ rod relative to its unit cell orientation.

For the Sn-0.7Cu/Cu joint, EBSD mapping showed multiple β-Sn crystal orientations and no evidence of solidification twinning (Fig. 9). A dendrite near the upper surface, highlighted by the insert, is growing almost in the sectioning plane and has a dendrite growth direction close to <110>, similar to the Sn-3.0Ag-0.5Cu/Cu joint in Fig. 8. The other insert confirms that the Cu_6_Sn_5_ rods are oriented along [0001]. The larger number of β-Sn orientations and wider range of misorientation angles in Fig. 9 than Fig. 8 is a significant difference between Sn-0.7Cu/Cu and Sn-3.0Ag-0.5Cu/Cu joints, that occurs both in these *in-situ* experiments and other BGA studies^30^,^37^.

In summary, using time-resolved synchrotron X-ray radiography adapted to mimic the paste reflow soldering process, nucleation events and microstructure evolution which cannot be deduced from post-mortem methods have been revealed and quantified during soldering solidification. The elucidation of solder joint microstructure development revealed in this study could be used as a basis for the design of an optimized and controlled microstructure in solder joints for future electronic interconnects technology.

## Methods

### Sample preparation

Sn-0.7Cu and Sn-3.0Ag-0.5Cu (wt.%) solder pastes with 35 μm average solder sphere diameter were used. A Cu plated printed circuit board (Fire retardant-FR4 type) with 600 μm ball pitch size was cross sectioned to produce a Cu-OSP substrate suitable for radiography with 100 μm thickness and 600 μm wide pitch. A small amount of solder paste (approximately 0.0002 g) was placed on the Cu pad in a cavity within a 100 μm thick polytetrafluoroethylene (PTFE) spacer sheet with an observation window of 10 × 10 mm^2^ and a vent for flux outgassing. Finally, the paste, substrate and PTFE were secured between two quartz plates. Further details, including a figure of the materials and sample preparation are given in the Supplementary Figure 2.

### Synchrotron X-ray Imaging

Experiments were conducted at BL20XU in the SPring-8 synchrotron using the solidification observation setup developed in previous research^14^,^39^ and an X-ray energy of 21 keV. Transmitted images were converted into visible light and recorded in a digital format with 2,000 × 2,000 pixels representing a 1 mm X 1 mm field of view giving a resolution of 0.477 μm per pixel. An exposure time of 120 ms per frame with 20 frames per second was used. A radiation furnace with graphite heating elements applied a reflow profile that heated from room temperature to approximately 250 °C at 0.33 °C/s, held at this peak temperature for 30 s before cooling down at approximately 0.33 °C/s.

### X-ray image processing and analysis

Image sequences were flatfield corrected and normalized against 10 frames shortly before the nucleation of primary intermetallic and flux voids for the primary intermetallic study and void study respectively using Image-Pro Plus v.7.0. For the study of primary intermetallics, a 3 × 3 × 5 (x,y,t) median filter was applied and for the study of tin dendrites, a 3 × 3 (x,y) median filter was applied. To quantify the growth of flux voids, Image-Pro Plus v.7.0 was used for object tracking. To quantify the primary Cu_6_Sn_5_ solidification kinetics, a routine was written in MATLAB 7.1 to identify the time (frame) at which each pixel becomes a solid pixel. First, the transmitted X-ray intensity was smoothed in time to reduce noise using a locally weighted linear regression. The sudden decrease in the intensity associated with a liquid pixel becoming a solid pixel was defined as the intersection of a linear-fit line to the flat region prior to solidification with a linear-fit line through the point with steepest decreasing slope. Crystal growth kinetics were then extracted from the solidification time of each pixel within each Cu_6_Sn_5_ crystal.

### Analytical Scanning Electron Microscopy (SEM)

Beamline samples were polished for scanning electron microscopy (SEM) and electron backscatter diffraction (EBSD) analysis. For measuring the dendrite arm spacing, polished samples were lightly etched (93% distilled water +5% sodium hydroxide +2% 2−nitrophenol). A Zeiss Auriga field emission gun SEM was used, with an Oxford Instruments INCA 80 mm^2^ x-sight energy dispersive X-ray (EDX) detector and a Bruker EBSD detector. EBSD mapping was conducted at 20 kV, scanning at 1 μm step per pixel and 50 ms exposure time. Kikuchi patterns were analysed using Bruker Espirit 2.0 software.

## Additional Information

**How to cite this article**: Mohd Salleh, M. A. A. *et al*. In situ imaging of microstructure formation in electronic interconnections. *Sci. Rep.* **7**, 40010; doi: 10.1038/srep40010 (2017).

**Publisher's note:** Springer Nature remains neutral with regard to jurisdictional claims in published maps and institutional affiliations.

## Supplementary Material

Supplementary Data and Methods

## References

[b1] Boettinger W. J. . Solidification microstructures: recent developments, future directions. Acta Materialia 48, 43–70, doi: http://dx.doi.org/10.1016/S1359-6454(99)00287-6 (2000).

[b2] Arfaei B. & Cotts E. Correlations Between the Microstructure and Fatigue Life of Near-Eutectic Sn-Ag-Cu Pb-Free Solders. Journal of Electronic Materials 38, 2617–2627, doi: 10.1007/s11664-009-0932-y (2009).

[b3] Liu W. & Lee N.-C. The effects of additives to SnAgCu alloys on microstructure and drop impact reliability of solder joints. JOM 59, 26–31, doi: 10.1007/s11837-007-0085-5 (2007).

[b4] Lu M., Shih D.-Y., Lauro P., Goldsmith C. & Henderson D. W. Effect of Sn grain orientation on electromigration degradation mechanism in high Sn-based Pb-free solders. Applied Physics Letters 92, 211909, doi: http://dx.doi.org/10.1063/1.2936996 (2008).

[b5] Huang M. L. & Yang F. Size effect model on kinetics of interfacial reaction between Sn-xAg-yCu solders and Cu substrate. Scientific Reports 4, 7117, doi: 10.1038/srep07117 (2014). 25408359 PMC4236743

[b6] Frear D. R. Issues related to the implementation of Pb-free electronic solders in consumer electronics. Journal of Materials Science: Materials in Electronics 18, 319–330, doi: 10.1007/s10854-006-9021-7 (2006).

[b7] Waldrop M. M. The chips are down for Moore’s law. Nature 530, 144–147 (2016). 26863965 10.1038/530144a

[b8] Hsiao H.-Y. . Unidirectional Growth of Microbumps on (111)-Oriented and Nanotwinned Copper. Science 336, 1007–1010, doi: 10.1126/science.1216511 (2012). 22628648

[b9] Nguyen-Thi H. . On the interest of synchrotron X-ray imaging for the study of solidification in metallic alloys. Comptes Rendus Physique 13, 237–245, doi: http://dx.doi.org/10.1016/j.crhy.2011.11.010 (2012).

[b10] Mathiesen R. H., Arnberg L., Mo F., Weitkamp T. & Snigirev A. Time Resolved X-Ray Imaging of Dendritic Growth in Binary Alloys. Physical Review Letters 83, 5062–5065 (1999).

[b11] Li B., Brody H. D. & Kazimirov A. Real Time Synchrotron Microradiography of Dendrite Coarsening in Sn-13 Wt Pct Bi Alloy. Metall and Mat Trans A 38, 599–605, doi: 10.1007/s11661-006-9058-5 (2007). 15697414

[b12] Zeng G. . Solidification of Sn-0.7Cu-0.15Zn Solder: *In Situ* Observation. Metall and Mat Trans A 45, 918–926, doi: 10.1007/s11661-013-2008-0 (2014).

[b13] Yasuda H. . Direct observation of stray crystal formation in unidirectional solidification of Sn–Bi alloy by X-ray imaging. Journal of Crystal Growth 262, 645–652, doi: http://dx.doi.org/10.1016/j.jcrysgro.2003.09.052 (2004).

[b14] Gourlay C. M. . *In situ* investigation of unidirectional solidification in Sn–0.7Cu and Sn–0.7Cu–0.06Ni. Acta Materialia 59, 4043–4054, doi: http://dx.doi.org/10.1016/j.actamat.2011.03.028 (2011).

[b15] Zhu J. . Real time observation of equiaxed growth of Sn–Pb alloy under an applied direct current by synchrotron microradiography. Materials Letters 89, 137–139, doi: http://dx.doi.org/10.1016/j.matlet.2012.08.094 (2012).

[b16] Zhou P. . *In situ* study on growth behavior of Cu6Sn5 during solidification with an applied DC in RE-doped Sn–Cu solder alloys. Journal of Materials Science: Materials in Electronics 25, 4538–4546, doi: 10.1007/s10854-014-2201-y (2014).

[b17] Mohd Salleh M. A. A., McDonald S. D., Yasuda H., Sugiyama A. & Nogita K. Rapid Cu6Sn5 growth at liquid Sn/solid Cu interfaces. Scripta Materialia 100, 17–20, doi: http://dx.doi.org/10.1016/j.scriptamat.2014.11.039 (2015).

[b18] Mohd Salleh M. A. A. . Effect of Ni on the Formation and Growth of Primary Cu6Sn5 Intermetallics in Sn-0.7 wt.%Cu Solder Pastes on Cu Substrates During the Soldering Process. Journal of Electronic Materials 45, 154–163, doi: 10.1007/s11664-015-4121-x (2015).

[b19] Ma H. T. . *In-situ* study on growth behavior of Ag3Sn in Sn–3.5Ag/Cu soldering reaction by synchrotron radiation real-time imaging technology. Journal of Alloys and Compounds 537, 286–290, doi: http://dx.doi.org/10.1016/j.jallcom.2012.05.055 (2012).

[b20] Huang M. L., Yang F., Zhao N. & Yang Y. C. Synchrotron radiation real-time *in situ* study on dissolution and precipitation of Ag3Sn plates in sub-50 μm Sn–Ag–Cu solder bumps. Journal of Alloys and Compounds 602, 281–284, doi: http://dx.doi.org/10.1016/j.jallcom.2014.03.047 (2014).

[b21] Qu L., Zhao N., Zhao H. J., Huang M. L. & Ma H. T. *In situ* study of the real-time growth behavior of Cu6Sn5 at the Sn/Cu interface during the soldering reaction. Scripta Materialia 72–73, 43–46, doi: http://dx.doi.org/10.1016/j.scriptamat.2013.10.013 (2014).

[b22] Qu L., Ma H. T., Zhao H. J., Kunwar A. & Zhao N. *In situ* study on growth behavior of interfacial bubbles and its effect on interfacial reaction during a soldering process. Applied Surface Science 305, 133–138, doi: http://dx.doi.org/10.1016/j.apsusc.2014.03.003 (2014).

[b23] Kotadia H. R., Howes P. D. & Mannan S. H. A review: On the development of low melting temperature Pb-free solders. Microelectronics Reliability 54, 1253–1273, doi: http://dx.doi.org/10.1016/j.microrel.2014.02.025 (2014).

[b24] Lee T.-K., Bieler T. R., Kim C.-U. & Ma H. In Fundamentals of lead-free solder interconnect From Microstructure to Reliability. Ch. Microstructure Development: Solidification and Isothermal Aging, 95–96 (Springer, 2015).

[b25] Anderson I. E. Development of Sn–Ag–Cu and Sn–Ag–Cu–X alloys for Pb-free electronic solder applications. Journal of Materials Science: Materials in Electronics 18, 55–76, doi: 10.1007/s10854-006-9011-9 (2007).

[b26] Ho C. E., Tsai R. Y., Lin Y. L. & Kao C. R. Effect of Cu concentration on the reactions between Sn-Ag-Cu solders and Ni. Journal of Electronic Materials 31, 584–590, doi: 10.1007/s11664-002-0129-0 (2002).

[b27] Nogita K., Mu D., McDonald S. D., Read J. & Wu Y. Q. Effect of Ni on phase stability and thermal expansion of Cu6−xNixSn5 (X = 0, 0.5, 1, 1.5 and 2). Intermetallics 26, 78–85, doi: http://dx.doi.org/10.1016/j.intermet.2012.03.047 (2012).

[b28] Assael M. J. . Reference Data for the Density and Viscosity of Liquid Copper and Liquid Tin. Journal of Physical and Chemical Reference Data 39, 033105, doi: http://dx.doi.org/10.1063/1.3467496 (2010).

[b29] Thermo-Calc. TCSLD Database version 3.0 (2015).

[b30] Gourlay C. M., Belyakov S. A., Ma Z. L. & Xian J. W. Nucleation and Growth of Tin in Pb-Free Solder Joints. JOM 67, 2383–2393, doi: 10.1007/s11837-015-1582-6 (2015).

[b31] Kurz W. & Fisher D. J. Fundamentals of solidification. (Trans Tech Publications, 1992).

[b32] Pang J. H. L. Lead Free Solder: Mechanics and Reliability. (Springer, New York, 2011).

[b33] Tian Y. . Effect of intermetallic compounds on fracture behaviors of Sn3.0Ag0.5Cu lead-free solder joints during *in situ* tensile test. Journal of Materials Science: Materials in Electronics 23, 136–147, doi: 10.1007/s10854-011-0538-z (2012).

[b34] Arfaei B., Kim N. & Cotts E. J. Dependence of Sn Grain Morphology of Sn-Ag-Cu Solder on Solidification Temperature. Journal of Electronic Materials 41, 362–374, doi: 10.1007/s11664-011-1756-0 (2011).

[b35] Padilla E., Jakkali V., Jiang L. & Chawla N. Quantifying the effect of porosity on the evolution of deformation and damage in Sn-based solder joints by X-ray microtomography and microstructure-based finite element modeling. Acta Materialia 60, 4017–4026, doi: http://dx.doi.org/10.1016/j.actamat.2012.03.048 (2012).

[b36] Mu D. K., McDonald S. D., Read J., Huang H. & Nogita K. Critical properties of Cu6Sn5 in electronic devices: Recent progress and a review. Current Opinion in Solid State and Materials Science 20, 55–76, doi: http://dx.doi.org/10.1016/j.cossms.2015.08.001 (2016).

[b37] Lehman L. P., Xing Y., Bieler T. R. & Cotts E. J. Cyclic twin nucleation in tin-based solder alloys. Acta Materialia 58, 3546–3556, doi: http://dx.doi.org/10.1016/j.actamat.2010.01.030 (2010).

[b38] Yang S., Tian Y. & Wang C. Investigation on Sn grain number and crystal orientation in the Sn–Ag–Cu/Cu solder joints of different sizes. Journal of Materials Science: Materials in Electronics 21, 1174–1180, doi: 10.1007/s10854-009-0042-x (2009).

[b39] Mohd Salleh M. A. A., McDonald S. D., Yasuda H., Sugiyama A. & Nogita K. Rapid Cu6Sn5 growth at liquid Sn/solid Cu interfaces. Scripta Mater 100, 17–20 (2015).

