# Supplementary material for: In situ imaging of microstructure formation in electronic interconnections
Source: Sci Rep. 2017 Jan 12;7:40010. doi: 10.1038/srep40010 (PMC5227712; doi:10.1038/srep40010)
Supplement: Supplementary Data and Methods [file srep40010-s1.pdf]

In situ imaging of microstructure formation in electronic interconnections

M.A.A. Mohd Salleh[1,2,3*], C.M. Gourlay[3], J.W. Xian[3], S.A. Belyakov[3], H. Yasuda[4], S.D. McDonald[1], K. Nogita[1]

[1]Nihon Superior Centre for the Manufacture of Electronic Materials (NS CMEM), School of Mechanical and Mining Engineering, The University of Queensland, 4072 St Lucia, Queensland, Australia.

[2]Centre of Excellence Geopolymer and Green Technology, School of Materials Engineering, Universiti Malaysia Perlis (UniMAP), Taman Muhibbah 02600, Jejawi, Arau, Perlis, Malaysia.

[3]Department of Materials, Imperial College, London SW7 2AZ, United Kingdom.

[4]Department of Materials Science and Engineering, Kyoto University, Sakyo-ku, Kyoto 606-8501, Japan.

Manuscript title: In situ imaging of microstructure formation in electronic interconnections

Authors: M. A. A. Mohd Salleh[*], C.M. Gourlay, J.W. Xian, S.A. Belyakov, H. Yasuda, S. D. McDonald, K. Nogita.

Supplementary Data

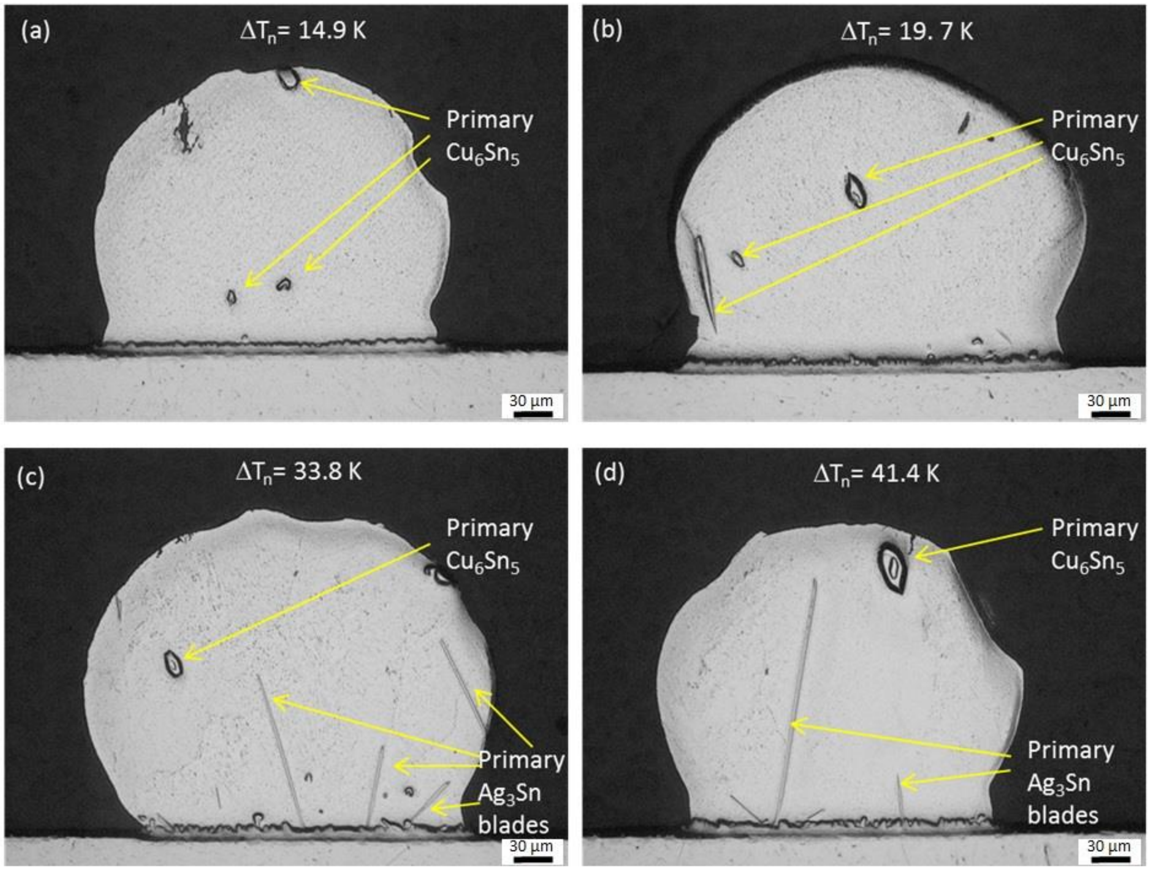

Supplementary Figure 1: SEM-BSE images of Sn-3.0Ag-0.5Cu/Cu joints at undercooling of (a) 14.9 K, (b) 19.7 K, (c) 33.8 K and (d) 41.4 K, indicating primary Ag_3Sn blades only formed in the joints at undercoolings higher than 20 K.

Authors: M. A. A. Mohd Salleh*, C.M. Gourlay, J.W. Xian, S.A. Belyakov, H. Yasuda, S. D. McDonald, K. Nogita.

Supplementary Methods

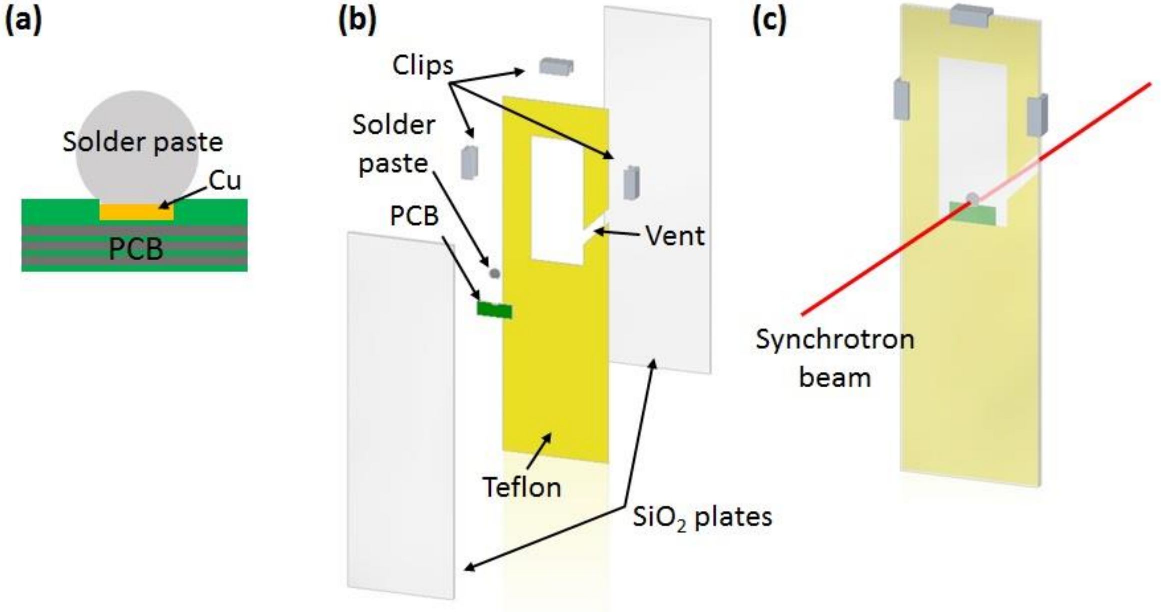

Supplementary Figure 2: Schematic diagram of in situ soldering observation experiment sample cell setup. (a) sample of solder paste on Cu PCB (b) parts of sample cell and (c) assembled parts of sample cell. Note that image is not to scale.

Solder pastes with 35 µm average solder sphere diameter were used with similar weight (approximately 0.0002 g) were used throughout the experiments. A Cu plated with organic soldering preservative (OSP) surface finish printed circuit board (Fire retardant-FR4 type) with 600 µm ball pitch size was cross sectioned to produce a Cu-OSP substrate suitable for radiography with 100 µm thickness. A small amount of solder paste was placed on the Cu pad (Supplementary Figure 2a). As in Supplementary Figure 2b and 2c, samples were placed in a cavity within a 100 µm thick polytetrafluoroethylene (PTFE) spacer sheet with an observation

window of 10 x 10 mm^2 and a vent for flux outgassing. Finally, the paste, substrate and PTFE were secured between two quartz plates.
